# Distributions of Silica and Biopolymer Structural Components in the Spore Elater of *Equisetum arvense*, an Ancient Silicifying Plant

**DOI:** 10.3389/fpls.2019.00210

**Published:** 2019-03-05

**Authors:** Victor V. Volkov, Graham J. Hickman, Anna Sola-Rabada, Carole C. Perry

**Affiliations:** Interdisciplinary Biomedical Research Centre, School of Science and Technology, Nottingham Trent University, Nottingham, United Kingdom

**Keywords:** *Equisetum*, spore, microscopy, Raman, silica, DFT

## Abstract

*Equisetum* species are primitive vascular plants that benefit from the biogenesis of silica bio-organic inclusions in their tissues and participate in the annual biosilica turnover in local eco-systems. As means of *Equisetum* reproduction and propagation, spores are expected to reflect the evolutionary adaptation of the plants to the climatic conditions at different times of the year. Combining methods of Raman and scanning electron microscopy and assisted with density functional theory, we conducted material spatial-spectral correlations to characterize the distribution of biopolymers and silica based structural elements that contribute to the bio-mineral content of the elater. The elater tip has underlying skeletal-like structural elements where cellulose fibers provide strength and flexibility, both of which are necessary for locomotion. The surface of the elater tips is rich with less ordered pectin like polysaccharide and shows a ridged, folded character. At the surface we observe silica of amorphous, colloidal form in nearly spherical structures where the silica is only a few layers thick. We propose the observed expansion of elater tips upon germination and the form of silica including encapsulated biopolymers are designed for ready dispersion, release of the polysaccharide-arginine rich content and to facilitate silica uptake to the developing plant. This behavior would help to condition local soil chemistry to facilitate competitive rooting potential and stem propagation.

## Introduction

The occurrence of silica in algae (i.e., diatoms) (Brunner et al., 2009), simple animals (i.e., sponges) (Mann et al., 1983) and in plants (Sachs, 1862; Lewin and Reimann, 1969; Page, 1972; Hodson et al., 2005) are important examples of bio-mineralization in evolution. Silica may accumulate in pith (stem), cortex (stem or root), mesophyll (leaves) and vascular tissues of plants (Lewin and Reimann, 1969). *Equisetum spp*. (commonly known as horsetail) are ancient examples of living vascular plants (Page, 1972). It has been reported that *Equisetum spp*. take up mono-silicic acid from the soil to accumulate silica in their tissues (Timell, 1964; Grégoire et al., 2012). The genus benefits from a broad global distribution and yet can also be considered as an invasive and persistent weed.

Electron microscopy of silica depositions in epidermal cells of an *Equisetum sp*. (Kaufman et al., 1971) provided strong support for the earlier expectations (Lewin and Reimann, 1969) that silica may serve to (1) provide mechanical strength and rigidity of cellular wall and/or of tissue (Perry and Fraser, 1991; Hodson et al., 2005; Grégoire et al., 2012), (2) prevent excessive water loss through the epidermis (Gao et al., 2004), and (3) protect against pathogens and predators (Stahl, 1888; Guerriero et al., 2018; Coskun et al., 2019). Indeed, recently, correlation between the localization of silica and callose (a type of polysaccharide in plants) was reported which allowed the suggestion of a unique relationship between uptake of silicic acid and depositions of biogenic silica and callose, which were considered to provide resistance against fungal infection in horsetail (Guerriero et al., 2018) though alternative hypotheses as to how resistance is achieved have recently been published (Coskun et al., 2019).

Silica deposited in living organisms, often referred to as biogenic silica or biosilica, is generally accepted to be in amorphous forms (Perry et al., 1984; Brunner et al., 2009; Neethirajan et al., 2009). Silicification of *Equisetum* and its spores is a complex but potentially useful model system with insights (i.e., optical properties) that could aid commercial applications (Neethirajan et al., 2009). The formation of amorphous biosilica through the biosilicification process in *Equisetum spp*. is notable in its divergence from crystalline inorganic silica, with characteristics that have been described as a xerogel (Holzhüter et al., 2003). The chemical and morphological characteristics of the biosilica influence the biocompatibility of the material, being remarkably less harmful than crystalline polymorphs both natural and man-made, though silica toxicity is an active area of research (Fruijtier-Pölloth, 2012; Murugadoss et al., 2017). Silica produced by plants presents ordered hierarchical porous structures giving this material interesting properties with possible applications both in industry and in medicine (Davis, 2002; Sola-Rabada et al., 2018).

To understand the role of silica in biology and survival strategies of *Equisetum*, it is necessary to correlate both the distribution of biosilica inclusions and chemical properties of the inorganic component (at the junction with bio-tissue) with plant biology and biochemistry. In the early 70s (Kaufman et al., 1971), it was reported that in *E. hyemale*, silica is uniformly distributed over and within the outer epidermal cell walls, while in *E. arvense*, silica is concentrated in discrete structures (knobs and rosettes) projecting from the outer epidermal walls. Even though both rosette and knob structures were reported to contain inorganic silica, the character of silica distribution and the chemical nature of such inorganic inclusions in such structures, as well as in particulate structures on the outside of elaters, may be very different (Duckett, 1970; Kaufman et al., 1971; Perry and Fraser, 1991). In our previous study on the subject, we characterized the distribution of silica in stem nodes, internodes, basal branches, distal and leaves of *E. arvense* as fibrillary, globular and sheet-like silica ultra-structures (Perry and Fraser, 1991). According to the results, we anticipated the inorganic component would assert mechanical strength and rigidity and discussed the possible role of the cellulose micro-fibrillary network to template some of the considered silica depositions.

**FIGURE 1:**
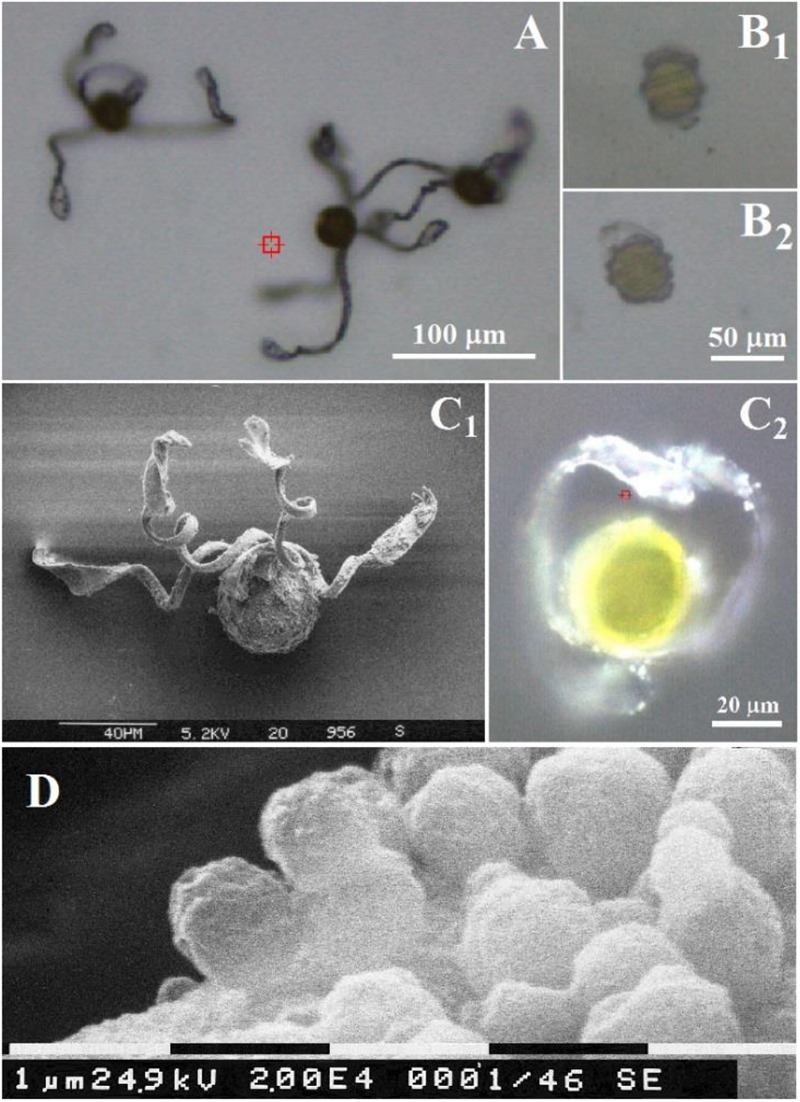
Optical microscopy images of *Equisetum* spores **(A)** under air and **(B)** in water. Scales bars are 100 and 50 μm, respectively. Comparison of an *Equisetum* spore under air by **(C_1_)** scanning electron microscopy and **(C_2_)** optical microscopy. Scales bars are 40 and 20 μm, respectively. **(D)** Scanning electron microscopy of spherical containers at surface of elater. Scale bar is 1 μm.

As a lower vascular plant species *Equisetum* reproduces with the aid of spores (Sadebeck, 1878; Newcombe, 1888; Beer, 1909; Erdtman, 1952), see Figure 1. Spores are notable for their complex morphology and motile nature. Beer (1909), Rudolf Beer reported that a “ripe spore contains a very considerable quantity of chlorophyll in its protoplast,” and that when “spores are heated with concentrated sulphuric acid on a cover-glass, very pretty siliceous skeletons are left behind.” Later spore protoplasm was reported to differentiate into a peripheral one, where storage substances are dominant, a middle zone containing chloroplasts, and an internal one surrounding the nucleus (Gullvag, 1968). The central body of a spore is approximately of 30–50 μm in diameter (Duckett, 1970). Each spore has four elaters, which respond to humidity variations (Newcombe, 1888).

Under dry air (depending on humidity) elaters can demonstrate periodic opening, and upon drying from a fully hydrated state, elaters may open rapidly to pitch a spore 1.5 mm above the surface (Marmottant et al., 2013). The described locomotion helps dispersal strategies using both hydrodynamics and aerodynamics because of local changes in environmental conditions. The structural mechanism of elater mobility is likely due to their bilayer structure: the inner layer consists of longitudinal microfibrils, the matrix of the outer elater formed because of structural granulations is expected to be less dense and porous (Uehara and Kurita, 1989). The differential volume change of one layer with respect to the other (Elbaum et al., 2007; Reyssat and Mahadevan, 2009) is suggested to be responsible for the curvature changes of the elaters (Marmottant et al., 2013). Besides a role in dispersion and spore motility, one may anticipate that due to the terminating paddle structure, elaters will have other purposes either when folded or when unfolded. The morphology of the paddles, structuring and elemental distributions at their surface (Perry, 1989) may suggest that in a dry environment paddles may assist the physical fixation of a spore at a site and conditioning of local biochemistry prior to germination.

In one of our earlier studies, we pioneered the characterization of silica distribution in elaters of *E. arvense* spores (Perry, 1989). On the surfaces of the spores and elaters, we identified small rounded objects ca. 500 nm in diameter protected by a coating of small silica particles, that contained proteinaceous material rich in arginine, low in aromatic amino acids and with a small amount of glucose containing polysaccharide material (Perry, 1989). In a relatively recent study, fluorescence labeling was used to observe “punctate” deposits of silica on the spore surfaces (Law and Exley, 2011). To understand the role of silica deposits on the surface of the spore/elater germination machinery we need to provide chemical/biological spatial-functional correlations between the bio-organic and bio-inorganic components. Correlations in chemical composition for different extractions (Currie and Perry, 2009) lent support to the idea that the observed spatial co-distributions of the organic and inorganic components are according to the genetically programmed biochemistry of the plant. However, a deeper insight would require characterization of the structural states of the silica and the organic moieties at the junctions. This is where, due to resolution and chemical selectivity, the methods of Raman microscopy sampling are particularly valuable. For example, the technique has been applied to the analysis of the chemical composition of *Equisetum hyemale*, where Gierlinger et al. (2008) described the non-uniform distribution of silica below a cuticular wax layer.

The challenge of what is being attempted becomes obvious if we compare the results by Raman microscopy with the conclusions of Kaufman et al. (1971) who used electron microscopy to report that silica is uniformly distributed over and within the epidermal surface in *Equisetum hyemale*. The differences, however, may be accounted for by variations in sample preparation (if the plane of cuts were explored or surfaces) and due to differences in penetration depth for different frequencies of radiation: being either a few or several hundreds of nanometers for X and Y, respectively. From this perspective, the results of the two techniques are both relevant and should be discussed comparatively if possible.

In this article we combine Raman microscopy with scanning electron microscopy (SEM) and elemental analysis to explore (and correlate) structural, vibrational and elemental properties on the paddle structure of a selected elater of a spore of *Equisetum arvense*. Further, we use computational studies of structures representing the major classes of materials anticipated to confirm identification of the materials present. The structure of the results section of the article is as follows: after describing the spore elater complex we present, (a) experimental spectra obtained from different regions of the system studied, (b) theoretical vibrational spectra calculated for representative (bio)chemical markers necessary for the reconstruction of Raman microscopy images, and (c) Raman difference microscopy images reconstructed for the selected spectral markers where for this task we adopt an ansatz for renormalization of Raman intensities. The described details on the distribution of bio-inorganic structural components allow us to discuss the bio-functionality of these structures and to hypothesize on survival strategies.

## Materials and Methods

Spore heads of a field horsetail, *Equisetum arvense*, were collected in Nottinghamshire (May 2018), see details in the Supporting Information.

Silica nanoparticles were synthesized by a modified Stöber method (Stober et al., 1968). The synthesized particles were rehydrated several times in the presence of deuterium oxide at high-temperature and vacuum avoiding annealing to allow for solvent exchange with deuterium oxide. For the optical studies 200 ± 12 nm diameter particles were used, the sizes of which were determined by employing dynamic light scattering (Zetasizer Nano-ZS, Malvern Instruments, Malvern, United Kingdom).

Material and elemental analysis: sample imaging and energy-dispersive X-ray spectroscopy (EDS) were conducted using a JEOL 7100FEG SEM equipped with an Oxford Instruments X-MaxN 80 mm^2^ EDS. Samples were mounted on an aluminum stub with carbon tape (TAAB, Aldermaston, United Kingdom). The instrument was operated in secondary electron mode with a 1.0 kV accelerating voltage for imaging with a beam of about 2 nm diameter. For elemental analysis (EDS), the accelerating voltage was set to 10.0 kV. Micrographs were collected and exported using PC-SEM v. 5.1.0.6 and EDS spectra were collected, processed and exported using Aztec 3.3 SP1.

Raman spectral studies were conducted using a DXR microscope from Thermo Fisher Scientific, Madison, WI, United States equipped with 50× and 100× microscopy objectives. The spectral resolution in the Raman experiment was down to 2 cm^-1^ according to the instrumental limit of the microscope operated with a 25 micron confocal slit or pinhole. The former was used for Raman spectral measurements when spatial resolution was not considered, while the latter was used for sampling of Raman maps. Raman measurements were made using 532 nm excitation radiation of 2 mW.

**FIGURE 2:**
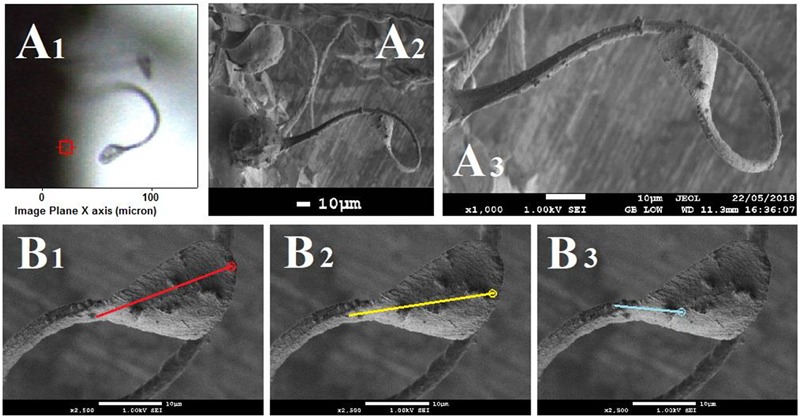
**(A)** Optical microscopy, **(A_1_)** and SEM images, **(A_2_,A_3_)** of an elater selected for simultaneous Raman microscopy; and **(B)** high resolution SEM images of the selected elater with lines red **(B_1_)**, yellow **(B_2_)**, and cyan **(B_3_)**, corresponding to the abundances of carbon, oxygen and silica, respectively; circles mark the destination points. Supporting Information provides the original (low resolution) SEM images with lines, along which the abundances of atoms were sampled.

To correlate results of SEM and Raman microscopy studies, in the latter we used a spore sample on the same aluminum stub fixed with double-sided carbon adhesive tape as prepared for SEM. However, since Raman studies cannot be conducted on samples deposited on carbon tape (due to immediate thermal degradation of carbon under laser 532 nm radiation even when working at minimal power), it was necessary to search for an elater that would be free and hanging from the edge of the carbon tape. Hence, upon Raman mapping with a short focal length 100× microscope objective, the elater would not be physically disturbed by the objective. Hereafter, every time a suitable elater was found, numerous pre-tests were conducted by taking Raman samples to monitor the mobility and structural stability of the elater. In particular, it was determined by periodic focusing of field radiation of different powers that the minimal possible power of 2 mW] could be used as under such the elater would stop changing the bending of its stem. After sampling Raman maps of different regions of a selected elater (and before the elater would demonstrate structural degradation and decomposition), we conducted SEM microscopy and elemental analysis for carbon, oxygen and silicon atoms on the same elater (as shown in Figure 2).

Raman activities at different sites of the selected elater were sampled with a spatial resolution of 1 micron in both directions of the imaging plane. As a Raman microscopy scan collects a set of spectra specific for defined positions in the image plane, in order to present Raman microscopy images specific to spectral signatures of interest, we must process each spectrum from the detected set to extract amplitudes of spectral components of interest. Being dependent on the nature of the vibration (normal) modes, abundance of contributing structural species, orientation and degree of orientational variance at the sampling sites, the extracted amplitudes therefore are informative on molecular structural distributions at interfaces. To approach this, we conducted reconstructions of Raman activity microscopy images (RAM) specific to selected vibrations according to:

(1) $$RAM\left(X,\;Y,\omega \right)\;=\sum_{\text{i}}\;A_{\omega ,i}\frac{1}{2\pi \sigma^{2}}\text{Exp}\left[-\frac{{\left(X-X_{\text{i}}\right)}^{2}}{2\sigma_{\text{x}}^{2}}-\frac{{\left(Y-Y_{\text{i}}\right)}^{2}}{2\sigma_{\text{y}}^{2}}\right]$$

where *i*, is the index of a site where a spectrum is taken, *A*_ω_,*_i_*, is the amplitude of the Raman resonance of interest in the detected spectrum, and ω, is the frequency of the resonance. Furthermore, the equation shows that we sum image projections of two-dimensional Gaussian source functions over all the defined sites *i*. Setting 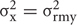 = 0.5μm^2^ provides the spatial full width of a source function. *X_i_* and *Y_i_* describe the position of the projection of the site *i* into the image plane. *X* and *Y* variables are sample distances from the site *i* in terms of the dimensions of detector pixels or displacements of a pinhole.

To utilize Raman microscopy imaging properly, we need to (1) understand “which” spectral resonance can be used to describe a particular structural species. In the last two decades, multivariate algorithms, such as principal component analysis (Pearson, 1901; Dieing and Ibach, 2011), independent component analysis (Ans et al., 1985; Hyvarinen et al., 2001) and methods of cluster analysis (Hedegaard et al., 2011), have gained popularity as powerful tools in the processing of microscopic images and Raman microscopy images of *Equisetum* tissues have previously been processed using principal component analysis (Gierlinger et al., 2008). In our studies, however, we approach Raman image reconstruction using (i) spectral markers obtained by exploring and comparing our experimental results, (ii) peak assignments previously reported in literature and, (iii) our predictions of quantum chemistry for the model molecular systems. The adopted approach is more computationally demanding than extraction of orthogonal (independent) spectral signatures upon application of linear algebra on detected spectral sets. However, this allows us, first, to avoid possible artifacts due to non-linear variances of Raman amplitudes as the surface of elaters in relation to molecular orientations are not trivial; and, second, trying to understand better the nature of the observed Raman responses.

**FIGURE 3:**
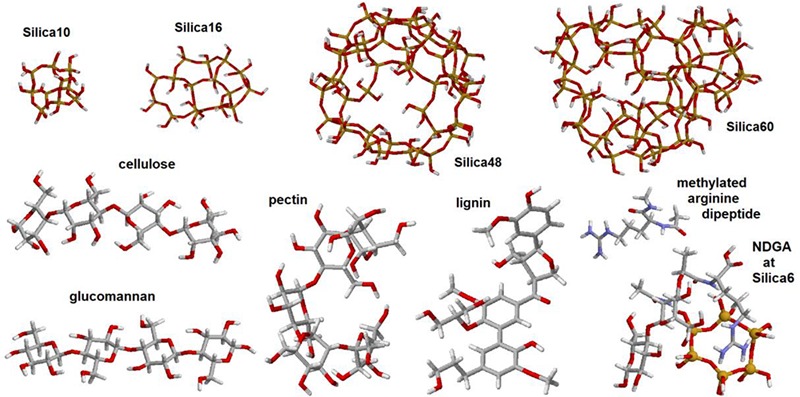
Graphical images of the structures used in quantum chemical density functional studies (DFT): silica-10 cage, silica-16 four-cycle cluster, silica-48 pored cage, silica-60 pored cage with a six-cycled double-layered structure, and model systems designed to represent structural components of cellulose, glucomannan, pectin, lignin, arginine dipeptide, and disaccharide with arginyl and acetyl-amine side groups coordinated with silica hexagonal cycle.

As we have mentioned, we adopted a computationally demanding approach to select responses suitable for molecular structural analysis using Raman image reconstructions. To manage the task, we conducted quantum chemical calculations for selected model systems using the 6–31 g^∗^ basis set and the restricted b3lyp functional (Becke, 1988; Lee et al., 1988) within the Gaussian 09 program package (Frisch et al., 2010). To model vibrational properties of inorganic structural components which may resemble that of the elater’s surface, we adopted (see Figure 3): (i) Silica-10, four hexagonal cycles merged in a cage system; (ii) Silica-16, four hexagonal cycles merged in a single layer system; (iii) Silica-48, single layer spherical structure with two pores on opposite sides; and (iv) Silica-60, single layer spherical structure, with two pores on opposite sides, fused with a cage to model a small span of a double layered system. To model vibrational properties of bio-organic components as expected in *Equisetum* tissues, we adopted structural segments of cellulose, glucomannan, pectin, lignin and a methylated dipeptide with arginine side group and arginyl-n-acetyl-di-glucosamine (NDGA) at a Silica-6 hexagonal cycle. Here, we used the following structural definitions, as described in the literature: cellulose is a linear polysaccharide consisting of thousands of β(1-4) linked D-glucose units (Updegraff, 1969), glucomannan is a hemicellulose polymer, with linearly linked β(1-4)-linked D-mannose and D-glucose in a ratio of 1.6:1 (Katsuraya et al., 2003), pectins are hetero-polysaccharides with chains and branches of α(1-4)-linked D-galacturonic acid (Ridley et al., 2001), lignins are bio-organic polymers composed of phenylpropanoids p-hydroxyphenyl, guaiacyl, and syringyl (Boerjan et al., 2003; Martone et al., 2009). The scaling factor for the calculated Raman dispersions was 0.97.

Using the results of quantum chemistry and experimental Raman spectroscopy to correlate the presence of different chemical species at the elater surface, we show differences between Raman microscopy images reconstructed for selected vibrations, while the intensities of the Raman images are scaled to be equal. This intensity renormalization allows qualitative (relative) characterization of spatial co-distributions of chemical species of interest at very complex interfaces and surfaces.

In the following Results section we describe: Raman spectral properties of a spore central body and of its elaters; review theoretically predicted spectral responses for the model molecular systems; select spectral markers and demonstrate the results of Raman difference microscopy images constructed for the selected spectral markers.

## Results

### Bright Field Optical Microscopy

The structural changes of spores of *E. arvense* upon humidity are shown in Figure 1. Under low humidity elaters are unfolded or partially unbent as shown in Figure 1A, whereas, in an aqueous environment (or in humid air), elaters wrap around the main spore body (see Figure 1B). The diameter of the central body of the spores corresponds well to the typical reported for this species, approximately 30–50 μm (Duckett, 1970). It is also observed, Figure 1C, that at the surfaces of the paddle structures and at the sides of the stems of the elaters there are small rounded objects, Figure 1D which have been previously reported to contain polysaccharide and proteinaceous material rich in arginine and reinforced with porous silica layers (Perry, 1989). Let us now describe the Raman spectral properties of a spore central body and of its elaters.

### Raman Responses From the Spore Central Body

**FIGURE 4:**
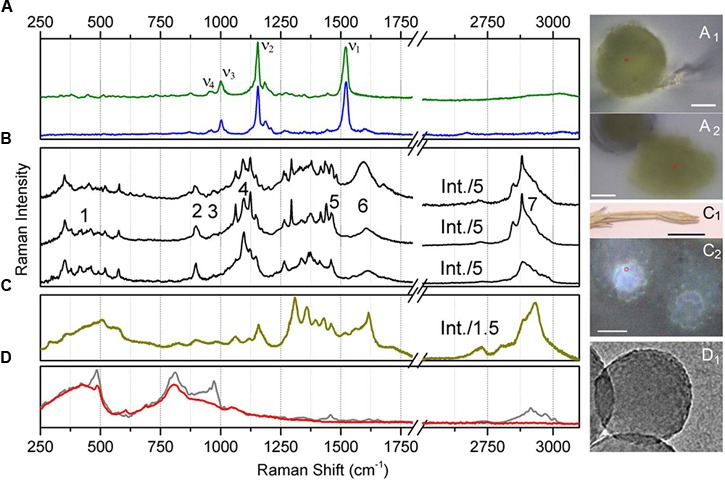
Raman spectra of *Equisetum* structures and silica nanoparticles. **(A)** Raman spectrum of the central body of an *Equisetum* spore in water (green line) and its optical microscopic image, **(A_1_)** (scale bar 2000 μm); and the Raman spectrum of the squeezed-out content of the central body of an *Equisetum* spore (blue line) and its optical microscopic image, **(A_2_)** (scale bar 10 μm). **(B)** Raman spectra sampled at different sites on the *Equisetum* spore elater. Numbers indicate spectral regions used for reconstructions of Raman microscopy images specific to these frequencies. **(C)** Raman spectra of silica rich rosette structures at the surface of the dried branch and its microscopic images, **(C_1_,C_2_)** (scale bars 10 and 20 μm, respectively). **(D)** Raman spectra of ca. 200 nm diameter amorphous silica nanoparticles (gray line) and the same sample after several hours of high temperature treatment at 1200 C (red line) and a TEM image **(D_1_)**.

Raman spectra sampled at the spore body under air and from the squeezed content are shown in Figure 4A. Accordingly, Figure 4A1,A2 show the corresponding optical microscopy images of the spore body under air and its content in water squeezed by application of a 300 μm glass cover slip. As expected, under 532 nm excitation, the detected spectra are dominated by spectral responses of carotenoid molecules which are generally present in light-harvesting proteins (Ruban et al., 1995, 2001). The spectra show the ν_1_ mode specific to C = C- stretching vibrations; ν_2_ mode of C-C stretches coupled either to C-H in-plane bending or C-CH_3_ stretching, the ν_3_ mode characteristic to CH_3_ in-plane rocking vibrations; and the ν_4_ mode specific to C-H out-of-plane bending (Rimai et al., 1973). The peak at 1520 cm^-1^ was fitted with two components, the major at 1523 cm^-1^ and the minor centered at 1515 cm^-1^. Accounting for the reported sensitivity of Raman on excitation wavelength (Ruban et al., 2001) and the possible linear regression (Gall et al., 2015) of the wavelengths of electronic transitions for zeaxanthin (Landrum and Bone, 2001), β-carotene (Britton, 1995), lutein (Takaichi and Shimada, 1992), and lutein epoxide (Melendez-Martinez et al., 2005), on the frequencies of the ν_1_ of C = C- stretching vibrations, we may anticipate that the main component fitted at 1523 cm^-1^ is due to the contribution of β-carotene. The smaller component is likely due to Fermi resonance with possible combinations and overtones. At the same time, it cannot be completely ruled out that the lower frequency component at 1515 cm^-1^ may be a signature of another carotenoid, like, for example, rhodoxanthin as was reported to be present in significant amounts in sporiferous shoots of *E. arvense* (Czeczuga, 1985). Accounting for dependencies on the wavelength of Raman excitations, the reported electronic and vibrational properties for rhodoxanthin (Chabera et al., 2009; Berg et al., 2013) may fit approximately the correlation electronic transitions on ν_1_ of the C = C- stretching frequency, as suggested in Boerjan et al. (2003).

### Raman Responses From Spore Elaters

Next, let us review the results of Raman spectroscopy of elaters, which, as organelles, have been described as four narrow spiral bands, formed upon division of the external coat of a spore at maturity (Sadebeck, 1878). Figure 4B shows several Raman spectra sampled at various sites on elater paddle structures. The spectra show two rich and complex subsets of Raman activity: between 250 and 750 cm^-1^ and between 870 and 1750 cm^-1^ which is expected to be due to both, inorganic and organic structural components (Sapei et al., 2007; Gierlinger et al., 2008). Also, in the spectral region covering 2600 to 3100 cm^-1^, resonances which are typical for -CH stretching modes of bio-organic molecules are observed (Atalla and Agarwal, 1985; Wiley and Atalla, 1987; Edwards et al., 1997; Agarwal and Ralph, 1997; Agarwal et al., 2001; Jahn et al., 2002; Synytsya et al., 2003). The spectra shown are site specific and are selected to demonstrate spectral diversity.

**FIGURE 5:**
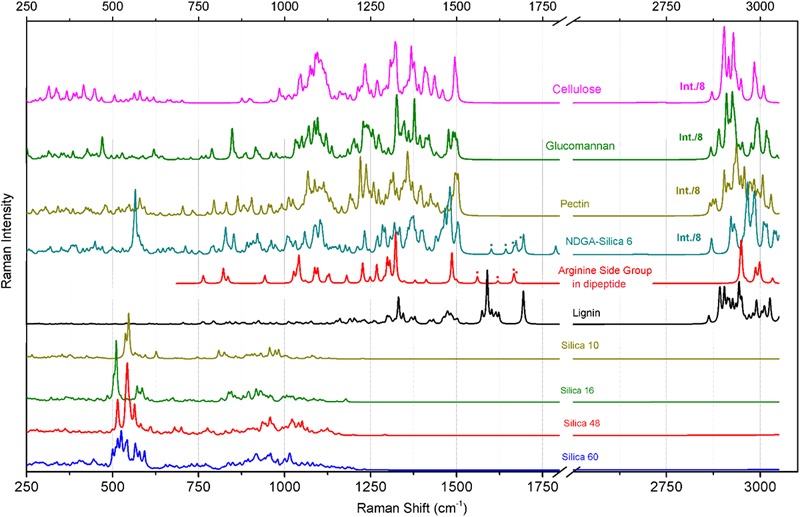
Raman responses calculated with DFT for a series of model systems designed to represent structural components of cellulose, glucomannan, pectin. arginine side group, and silica structures as indicated. The structures of the model systems are presented in Figure 3. The dotted marks denote spectral contributions of NH_2_ bending modes specific to the arginine side group in the considered systems.

To analyze the observed spectral responses, we use the results of computational quantum-chemical density functional theory (DFT) calculations to account for the spectral contributions of bio-organic and bio-mineral species we expect to be present: (1) silica structures, (2) carbohydrates, and (3) lignin(s), amino acids and polypeptides; as spectral markers to discuss the spatial distribution of the expected molecular species. In Figure 5 we present the calculated isotropic Raman responses (DFT predictions) for silica and hydro-carbon molecular structures, as shown in Figure 3, which we may consider as representatives of the main structural moieties present at the surface of the elater paddle structure.

#### Deduction of Spectral Markers Specific to Silica Structures

Raman responses of inorganic amorphous silica have been previously reported in literature. According to the results of early studies on Raman responses in silica gels (Gailliez-Degremont et al., 1997), attribution of (a) strong activities at 430–440 and 490–495 cm^-1^ to in-plane Si-O-Si vibrations and to the modes associated with SiO_4_ tetrahedra with a non-bridging oxygen atom; (b) medium and weak intensity signals at 800 and 1070 cm^-1^ to Si-O-Si symmetric and asymmetric stretching, respectively; and, (c) weak and medium responses at 910–920 and 970–980 cm^-1^ to surface and internal silanol stretching, respectively. These assignments were adopted to discuss the experimental results of Raman microscopy studies on the distribution of silica in a knob structure of *E. hyemale* (Gierlinger et al., 2008). In particular, Gierlinger et al. suggested that (i) an “overall remarkably high intensity” below 580 cm^-1^ should be a signature of amorphous silica in *Equisetum* tissue; (ii) Raman resonance at 802 cm^-1^ is due to Si–O–Si and Si–C stretching (Gailliez-Degremont et al., 1997); and (iii) the strong band at 973 cm^-1^ is specific to surface and internal silanol stretching. In a separate report (Sapei et al., 2007), the authors discussed the role of silica hydration in contact with polysaccharides to explain the presence of the peak at 973 cm^-1^. Note that this resonance was detected at the knob tip but not when the signal was sampled from a silica rich layer adjacent to epidermal cells.

To understand better the possible contributions of silica inclusions into the Raman spectra of elaters (Figure 4B), in Figure 4C we show Raman spectra from silica rich rosette structures at the side of an aged dry branch (the corresponding microscope images are also shown in Figure 4C_1_,C_2_); and in Figure 4D we show the Raman response from synthesized silica nanoparticles after different thermal treatments (TEM image is shown in Figure 4D_1_). The response from the latter systems is particularly helpful to verify the spectral contributions specific to Si-OH groups, which are expected at the particle surface (Perry and Keeling-Tucker, 2000). As it emerges from IR (Bunker et al., 1989; Morrow and McFarlan, 1992), Raman (Brinker et al., 1982; Humbert, 1995), and NMR (Chuang and Maciel, 1996) experimental studies, amorphous silica surfaces may undergo dehydroxylation via condensation of vicinal silanols upon heating. Consistently, Raman spectra in Figure 4D shows a significant decrease of Raman activity at 976 cm^-1^ for thermally treated amorphous silica particles compared to samples maintained at room temperature.

Inspection of the nature of the normal modes by DFT reveals that admixture of delocalized out-of-plane Si-OH bending and in-plane Si-O-Si symmetric vibrations of SiO_4_ tetrahedra contribute to the Raman activities anticipated at 500 cm^-1^, Figure 5. The variations in this spectral region are due to: (a) relative weights of the two types of vibrations, (b) geometry of hydrogen Si-OH…O-H bonding, which may not be optimal, (c) extent of delocalization, and (d) effects of coupling that contribute to excitonic splits. There is a noticeable tendency for the normal modes, where the contributions of out-of-plane Si-OH bending mode dominate, to manifest at the lower frequency side. The delocalized in-plane Si-OH bending modes are shown in the spectral region between 700 and 1200 cm^-1^. The diversity in this region is due to different degrees of admixing of such bending activities with O-Si-O symmetric stretching at about 800 cm^-1^, or with Si-O stretching at about 900 cm^-1^, or with Si-O-Si antisymmetric vibrations at 1000 cm^-1^ and above. The results of our calculations partially agree with assignments for silica gels (Gailliez-Degremont et al., 1997). However, DFT theory suggests a significant (if not a leading) role for various out-of-plane and in-plane Si-OH bending modes in the considered structures. This is certainly due to the enhanced contribution of the surface in small and single-double layered systems and this is what we expect at the surface of *Equisetum* elaters.

The calculated Raman spectral activities in the region between 700 and 1200 cm^-1^ for the silica structures (Figure 5) agree better with the experimental spectral responses from plant tissues, as shown in Figure 4A–C, rather than with spectral responses from the amorphous silica nanoparticles, as described in Figure 4D. Therefore, considering previous peak assignments, the spectral properties of silica nanoparticles and the suggestions of theoretical studies on silica cage systems, we may adopt the broad scattering intensity between 500–580 cm^-1^ as a signature of biosilica at the surface of an elater (marker #1 in Figure 4B). Also, we ascribe Raman activities in the spectral region 920–1000 cm^-1^, see marker #3 in Figure 4B, to biosilica. However, due to the weak intensity, we do not use such responses in our discussion based on Raman microscopy image reconstructions (see next section of the manuscript, Raman microscopy). Here, we wish to emphasize that we do not observe resonance at 805 cm^-1^ in the spectral response from elaters, while such a signal is strong in the response from the nanoparticles. It is interesting that both Raman signatures at 813 and 976 cm^-1^ are present in spectra detected form the rosette structures, though they are quite weak. The observed variances in the spectral responses of silica at the elater paddle structure suggest that structural composition of this mineral component at the surface of elaters may have a peculiar, distinct character, that likely differs from that found in amorphous inorganic nanostructures or in bio-organic deposits developed by plants for the purposes of defense and mechanical strength (Timell, 1964; Kaufman et al., 1971; Perry and Fraser, 1991; Hodson et al., 2005; Grégoire et al., 2012). This observation may have justifications from the perspective of spore biology – we will address this in our general discussion.

#### Deduction of Spectral Markers Specific to Carbohydrates

Polysaccharides are the main bio-organic structural components for plants and for *Equisetum spp*. tissues (Sapei et al., 2007; Gierlinger et al., 2008). To discuss our experimental observations, we adopt previous assignments for polysaccharide biopolymers such as cellulose (Wiley and Atalla, 1987; Edwards et al., 1997), glucomannan (Agarwal and Ralph, 1997), and pectin (Synytsya et al., 2003); as well as our predictions of Raman responses for the model systems, Figure 5. In these structural cases, theory predicts a set of spectrally narrow resonances in the frequency range 300–700 cm^-1^. C-OH out-of-plane bending contributes in this range but dominates at lower frequency. Symmetric and antisymmetric ring deformations with possible admixing of out-of-plane wagging of C-O-C bridges and C-OH out-of-plane bending contribute in the central region and at the high frequency side of this spectral range. In earlier studies, normal modes in this spectral region were mainly assigned to skeletal-bending modes involving the CCC, COC, OCC, and OCO internal coordinates of glucose-like moieties (Wiley and Atalla, 1987; Edwards et al., 1997). It is interesting to notice that for more regular (polycrystalline like) cellulose and glucomannan structures, normal modes in the frequency range of 300–700 cm^-1^ tend to group into higher and lower frequencies subsets with a window of relative transparency at 500 cm^-1^, which is where theory predicts that Raman resonances of silica shell systems would contribute the most, see the previous section. In the case of a more distorted pectin-like system, the out-of-plane C-OH bending modes admixed with the ring vibrations; fill the spectral range 300–700 cm^-1^ more uniformly.

Theoretical prediction (Figure 5) shows an absence of Raman activities for the more regular (polycrystalline) cellulose-like system in the spectral range between 700 and 1000 cm^-1^. For cellulose-like molecules, there are weak resonances at 870–900 cm^-1^ due to stretching/bending localized on C_4_-C_5_ and C_5_-C_6_ of glucopyranose rings. Besides this, the delocalized ring deformations which involve C_4_-C_5_-O and C_4_-O-C_1_ bending and C_1_-O stretching experience strong splitting at 723 and 989 cm^-1^. Calculations for glucomannan- and pectin-like systems provide similar results for this spectral range, but the normal modes are less delocalized due to the more deformed and less crystalline character of the materials. As a result, the C_2_-C_3_ and C_1_-O stretching, C_1_-C_2_-C_3_ symmetric and antisymmetric stretching, C_1_-O-C_4_ and C_1_-O-C_5_ symmetric stretching, and C_1_-O-C_4_ bending start to contribute in the spectral region between 700 and 1000 cm^-1^. In early studies, Raman activities in this spectral region were assigned to ν(COC) in plane symmetric mode at 897 cm^-1^ for glucomannans (Agarwal and Ralph, 1997; Gierlinger et al., 2008), to γ(COH)_ring_ for pectins at 817 and 832 cm^-1^, to a glycosidic asymmetric (COC) skeletal mode of α-anomers of pectins at 855 cm^-1^, and to α-glycosidic bonds of acidic pectin at 859 cm^-1^ (Synytsya et al., 2003). Considering the results of our theoretical studies we ascribe the experimentally detected Raman responses between 860–900 cm^-1^ (see marker #2 in Figure 4B) to local C_1_-O and C_2_-C_3_ stretching and C_1_-O-C_4_ bending modes of less regular and more deformed glucomannan- and pectin-like polysaccharides.

Comparing the experimental data with the results of DFT studies on polysaccharide model systems, we can state that the broad band between 1000 and 1150 cm^-1^ (Figure 4B) is dominated by delocalized C-O-C symmetric, C-O-C antisymmetric and C-O stretching admixed with some contributions from C-C stretching of the pyranose rings. The degree of delocalization is smaller for more deformed structures, like pectin. At lower frequencies, there are also contributions of partially delocalized pyranose rings corresponding to C-C stretching admixed with COH bending. The peak assignments are consistent with those reported in early studies for cellulose (Wiley and Atalla, 1987; Edwards et al., 1997), glucomannan (Agarwal and Ralph, 1997), and pectins (Synytsya et al., 2003). From our Raman microscopy studies, we attribute the contribution of Raman intensities between 1000 and 1150 cm^-1^ (marker #4 in Figure 4B) as a “generic” spectral signature of bio-organic components (mainly polysaccharides) for the normal modes, where interatomic displacements are mainly in the plane of pyranose rings. The next, higher energy spectral subsets of Raman activities centered at 1250 and 1375 cm^-1^ are specific to (possibly) delocalized C-CH bending modes associated with pyranose rings with contributions of both, C-CH and C-OH bending of the side groups, consistent with the early assignments reported in literature (Wiley and Atalla, 1987; Agarwal and Ralph, 1997; Edwards et al., 1997; Synytsya et al., 2003). It is interesting that for the three considered polysaccharides, our theory predicts (with minor spectral deviation) the CH_2_ scissor modes at around 1500 cm^-1^. In the experimental spectra this should correspond to spectral signatures at about 1464 cm^-1^, which we adopt as a spectral marker #5 in Figure 4B.

Finally, theoretical predictions for Raman activities specific to C-H stretching modes in the spectral range 2800–3010 cm^-1^ were investigated. In the case of the more regular cellulose and glucomannan structures, DFT anticipates (a) CH stretching of pyranose rings should dominate at the lower frequency side from 2870 to 2915 cm^-1^; (b) side group CH_2_ symmetric stretching mixed with CH activities of pyranose rings contribute mainly in the spectral range 2929–2949 cm^-1^; and, (c) side group CH_2_ antisymmetric stretching mixed with CH activities of pyranose rings would dominate at the higher frequency side from 2980 to 3009 cm^-1^. In comparison, theory predicts that CH Raman activities for less ordered pectin-like systems are broader. Inspection of the normal modes in that region reveals that in such systems, the vibrations with contribution of CH_2_ symmetric and antisymmetric stretching modes “explore” wider spectral ranges. From this perspective, the maximal Raman activity at 2882 cm^-1^, which we adopt as spectral marker #7 (Figure 4B) should be informative on the more ordered cellulose-like structural components (see Figure 3) at the interface of the elaters where CH_2_ antisymmetric stretching mixed with CH activities of pyranose rings dominate at the higher frequency side from 2980 to 3009 cm^-1^.

#### Deduction of Spectral Marker Specific to Lignin

Firstly, it is important to note that the intensities of the two Raman transitions at about 1607 and 1683 cm^-1^ (see Figure 4B) vary depending on the sampling spot at the surface of the elater paddle structure. Further, considering the spectral response from the rosette structures at the side of aged *Equisetum* dry branch, Figure 4C, two analogous resonances are observed, though the lower frequency resonance becomes very narrow and is shown at 1620 cm^-1^. According to previously reported results (Atalla and Agarwal, 1985; Agarwal et al., 2001; Jahn et al., 2002; Sapei et al., 2007), these two spectral signatures can be attributed to vibrations of the lignin structural component. Indeed, quantum calculations for the normal modes of a lignin-like segment (see Figure 5), suggest that the lower frequency resonance may be assigned to symmetric stretching in the aromatic ring, analogous to the ‘8a’ and ‘8b’ modes in substituted benzenes as assigned by Varsanyi (1974). The higher frequency resonance may be a signature of carbonyls which are present in lignin (Kirk and Farrell, 1987; Christopher et al., 2014).

To distinguish the role of lignin at the surface of elaters, we identified another independent spectral marker specific for this bio-polymer. For example, Figure 4C shows a dominant resonance at 2930 cm^-1^, which was reported as a spectral signature specific to lignin (Atalla and Agarwal, 1985; Agarwal et al., 2001; Jahn et al., 2002; Sapei et al., 2007). Considering that in the spectra detected from elaters, the Raman response at 2930 cm^-1^ is present as a smaller shoulder and it is not proportional to the spectral signatures at about 1607 and 1683 cm^-1^, we anticipate that lignin is a minor structural component at an elater surface, and, if present, its distribution is not uniform, and it is not well-ordered. According to the Raman response as shown in Figure 4C, this contrasts with the clear presence of lignin as a constituent structural component in a dried branch, where lignin fibers likely form well aligned depositions to contribute to mechanical stability. Accounting for both variances of experimental Raman responses (in Figure 4B,C) and DFT predictions for lignin in the spectral range between 1520 and 1750 cm^-1^, we adopt the experimentally observed Raman resonance at 1607 cm^-1^ as a representative signature for the lignin contribution and indicate it with marker #6 in Figure 4B.

#### Deduction of Spectral Markers Specific to Amino Acids and Polypeptides

The results of our previous studies (Perry, 1989) suggest that in this spectral region may be expected possible spectral signatures due to arginine functional groups and other protein related structural motifs. Exploring Raman activities predicted for the arginine side group, we confirm that there are two relatively weak and two strong in-plane NH_2_ bending modes for this moiety. These demonstrate variance in relative atomic displacements and in frequencies that depend on possible coordination of the side group with silica and orientation in respect to the backbone of a structural moiety: see the dotted markers next to the corresponding spectra in Figure 5. The results of theoretical studies suggest that the observed underlying spectrally broad background in the spectral range 1663–1754 cm^-1^ and observation at some sites of a relatively narrow spectral signature at 1675 cm^-1^ (see the upper spectrum in Figure 4B), may indicate the presence of arginine and carbonyl moieties, respectively. However, due to the weak intensity, breadth and non-systematic character of the spectral responses measured, we do not use these spectral signatures in our further analysis.

### Raman Difference Microscopy on the Bio-Inorganic Composition of an Elater Surface

**FIGURE 6:**
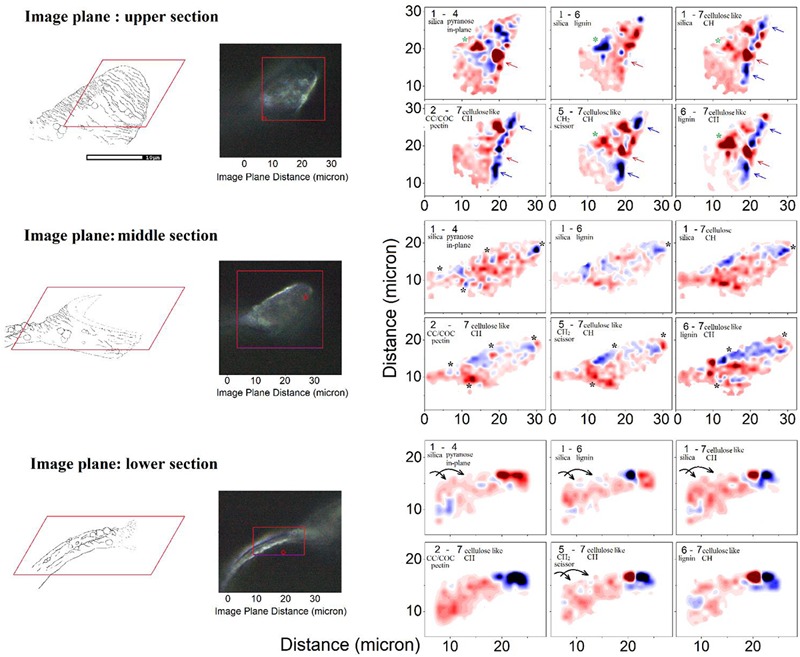
Raman microscopy of a single elater. Left: Graphical representations of the three levels at which the Raman map was obtained and the corresponding optical microscopy (100×) images of the mapped regions of the selected elater. Right: The Raman difference maps are calculated for the differences as indicated in the panels using spectral components as shown in Figure 4B. In the panels we provide brief details of the nature of considered spectral components. Detailed descriptions are provided in the main text. To explain the meaning of the color code, let us consider differences 2–7. There, blue areas indicate the spatial regions where the Raman activities of the subtracted Raman microscopy maps, reconstructed at frequency by marker #7 (see Figure 4), are larger than the Raman activities of the Raman microscopy maps reconstructed at frequency by marker #2. Red areas indicate spatial regions where the situation is the opposite of this.

Taking differences between equally scaled images (specific to the selected Raman activities) we gain a qualitative comparison of how one Raman active vibrational activity correlates or anti-correlates in respect to another in space. Accordingly, Figure 6 shows three sets of Raman microscopy maps corresponding to the upper, the middle and the lower sections of the same elater paddle structure. Here, we recapitulate the chemical-structural aspects we wish to stress using Raman difference maps. The Raman difference signal mapping labeled as 1–4, 1–6, and 1–7 would contrast spatial distributions of biosilica versus spatially aligned structural elements of generic extended cellulose-like polysaccharides mainly, lignin, and other generic organic contributions expressed through CH stretching, respectively. In contrast, the Raman microscope maps named 2–7, 5–7, and 6–7 would contrast spatial distribution of pectin-like polysaccharides (Synytsya et al., 2003; Gierlinger et al., 2008), δCH_2_ scissor modes of hydrocarbons and lignin versus distribution of organic contributions expressed through CH stretching.

**FIGURE 7:**
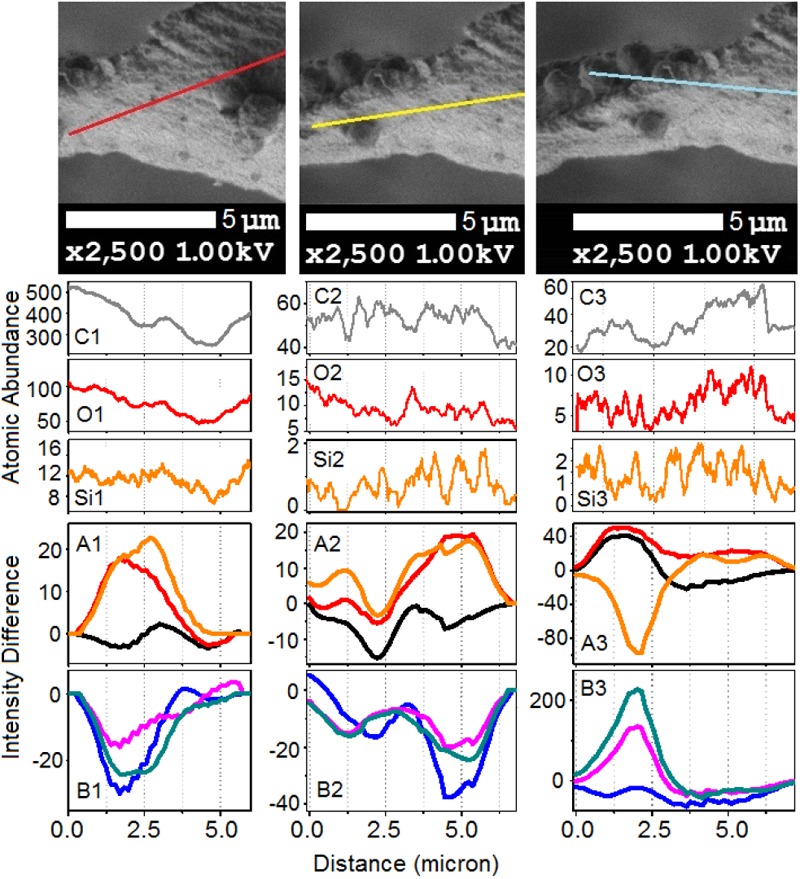
The upper set of images expands SEM images, as shown in Figure 2, featuring the bottom part of the paddle structure. **(C_1_,O_1_,Si_1_)** Abundances for carbon, oxygen, and silicon atoms along the sampling line as shown in the top set of images from left to right according to indexes i = 1–3, respectively. Panels **(A_1_,B_1_,A_2_,B_2_,A_3_,B_3_)** show Raman difference signals: 1–7 (black), 1–4 (red), 1–6 (orange), 2–7 (blue), 5–7 (magenta), and 6–7 (dark cyan) extracted from Raman difference images of the lower section, see Figure 6, along the lines as shown in the top set of images.

To gain deeper insight, in Figure 7, 8 we show Raman difference signals along selected directions of the lower and the upper sampling sections, as demonstrated in Figure 6. Note that, selected directions match those in Figure 2 where X-ray spectroscopy in the electron microscope was attempted. Considering the moderate accelerating voltage applied (to allow for the fragility of the specimen) and accounting for the approximate density of carbon atoms of about 24 atoms at 120Å^2^, we anticipate a relatively good sampling efficiency along line B_1_, but a loss of sampling efficiency when signals were detected along lines B_2_ and B_3_, see Figure 2. This is likely due to alteration in the orientation of the sampling plane, the gun and the detector during successive line scans. X-ray spectroscopy (EDS) instructs on the average relative levels of contributions of carbon and silicon atoms, and on the relative local variability. The former characteristic helps us understand the character of silica deposition. We see that, on average, the presence of silicon (as silica) is about 20 times smaller than that of the bio-organic component. This suggests the presence of thin (likely one or rarely few layers) clusters of silica at the interface. This agrees with the results of secondary ion mass spectrometry on some silica depositions in *Equisetum arvense* (Guerriero et al., 2018).

**FIGURE 8:**
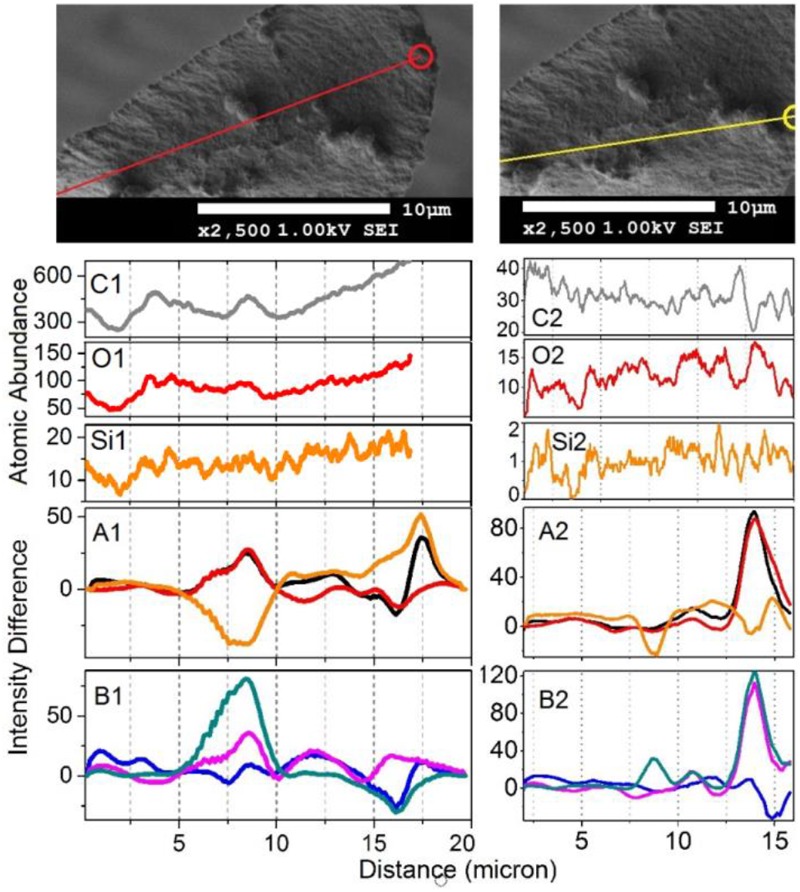
The upper set expands SEM images, as shown in Figure 2, featuring the middle part of the paddle structure. Explanations for the panels are the same as those in the caption of Figure 7, save the Raman difference signals: 1–7 (black), 1–4 (red), 1–6 (orange), 2–7 (blue), 5–7 (magenta), and 6–7 (dark cyan) are extracted from Raman difference images of the middle section, see Figure 6.

We first consider the bottom section of the elater, which comprises the connection between the stem and the paddle structure (Figure 6). This structural region has a very complex three-dimensional character, with a steep slope of the stem at the lower side. The EDS responses in Figure 7O1,C1,Si1 show the underlying trend - atomic abundances increase (from left to right). This is likely to be due to orientation effect – reflection toward detector is favorable at the slope. In the same spatial region, Raman difference signals 1–4, 1–6, and 1–7 in Figure 7A1 anti-correlate with the differences 2–7, 5–7, and 6–7 in Figure 7B1. For the biosilica component signals are collected more efficiently than those from the bio-organic structures and the extended cellulose-like contribution is prominent amongst the others. Comparatively, the atomic abundances (C3, O3, Si3) and the Raman difference signals maps (A3 and B3) as shown in Figure 7 are sampled from a more uniformly spatial region (structural components located at the same height), thus avoiding the tilting of the sampling plane. The SEM image shows that at the very left side of the sampling line, there is the presence of a spherical structure. Since, here, the sampling plane is not tilted; we may attribute the positive Raman differences 1–7 and 1–4 (in Figure 7A3) as signatures of silica surplus, likely due to the material of the vertical side walls of the structure. This is consistent with the fact that the positive signatures are spatially broader and “tipped” from the top. It is also interesting, that, in contrast to the dependences in Figure 7B1, the Raman differences 5–7 and 6–7 in Figure 7B3 in the spatial region of the spherical structure are positive and relatively narrower, comparing to differences 1–7 and 1–4 in Figure 7A3. This suggests that the deposition of pectin-glucomannan glycoside may be associated with that of silica, and that such polysaccharide components are likely imbedded in the bio-inorganic component/structure. Further, the lignin-like contributions are diminished at the center of the spherical structure (see orange line in Figure 7A3). Finally, the Raman difference signals maps shown in Figure 7A2,B2, reveal an intermediate character considering the above described cases. This can be explained as a spatial overlap of the areas where Raman signals are sampled. Note that, the physical limit of Raman microscopy resolution cannot be better than λ/2, and experimentally will always be inferior comparing to SEM. In Figure 8 are shown the atomic abundances and Raman difference signals in the upper section of the elater paddle structure. This section of the paddle structure is the most distant from the stem; however, similar tendencies to that of the lower section are observed when comparing Raman mapping (A1, B1 and A2, B2) with Figure 7A3,B3.

After exploring possible correlations of several Raman difference signals with mapping spatially parallelized with atomic abundances, it is time to review the two-dimensional Raman difference microscopy images (in Figure 6), to help unravel the morphogenetic plan of the *Equisetum* spore developed by millions of years of evolution. First, we consider the Raman difference map of the stem part, which is expected to provide mechanical stability and locomotion properties. Images specific to differences corresponding to Raman maps 1–4, 1–6, 1–7, and 5–7 indicate that silica deposition may have a spiral character, see arrows in the lower set of panels in Figure 6, and it may be coordinated with spatial distributions and orientation of CH moieties associated with a cellulose-like structural component.

Exploring the middle section Raman map, we may confirm that formation of spherical structures requires a morphogenetic plan requiring the embedding of polysaccharide and silica layers and a supply of material. To address this further, black and green stars have been placed on the images for the middle and the upper sections in Figure 6. For example, the lower black stars (of the middle section) indicate the region where silica is strongly associated with CH modes of cellulose-like component, but anti-correlates with a pectin-glucomannan-like fraction which is embedded inside the structure, more concretely in the center of a spherical structure. Similar trends are observed in the spatial region marked by green stars in the middle section.

Here, it is interesting to notice that SEM images of the upper part of the paddle structure indicate a more rigged and folded morphology of the surface at the tip. This is consistent with Raman microscopy maps. Raman maps reveal a more red-blue rapidly altering pattern toward the edge, and the very edge, apparently is reinforced with cellulose-like polycrystalline terminations – see the blue ridge at the edge in differences 1–7, 2–7, 5–6, and 6–7 in the Raman images specific to the upper part (see blue and red arrows in Figure 6).

Finally, it is necessary to address that in contrast to large scale preferences for distributions for pectin-glucomannan- and cellulose-like components reported in cells of stems and branches of *Equisetum spp*. (Perry and Fraser, 1991; Speck et al., 1998; Perry and Keeling-Tucker, 2003; Sapei et al., 2007; Gierlinger et al., 2008), distributions of polysaccharides and lignin at the elater paddle structure exhibits structure on the submicron scale. Overall, this correlates with the ridged-folded morphology of the surface of the elater paddle. This ridged-folded morphology becomes more obvious toward the edges.

## Discussion

**FIGURE 9:**
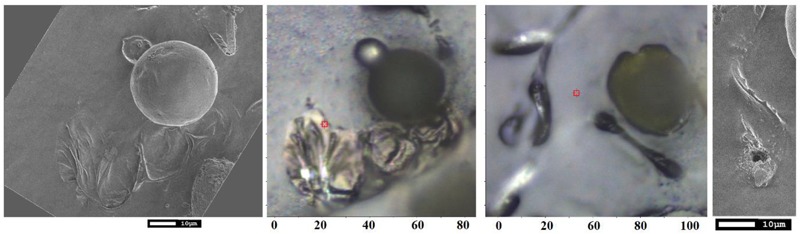
SEM and optical bright field microscopy images of spores on carbon tape for SEM studies 10 days after deposition.

Early TEM studies demonstrated that an elater can be found on the surface of the plasmodial plasma membrane as a thin belt-like structure spirally coiled around the middle layer (Uehara and Kurita, 1989). The structure consists of an inner granulo-fibrous zone and of an outer micro-fibrillar region which aligns parallel to the longitudinal axis. This structure provides structural heterogeneity of the inner and outer layers of the elaters in stems to demonstrate locomotion capacity by rapid differential volume change (Marmottant et al., 2013). In our studies, we address the complexity of bio-organic decoration of the paddle structure of the elater, which should be consistent with the needs of biological survival and propagation. Our SEM studies clearly indicate: (a) a ridged complex, of about 0.5 micron, at the elater’s surface, suggesting a folding complex nature of structuring of the bio-organic matter under the surface; (b) the presence of sub-micron diameter spherical structures on the ridged surface of the paddle structure and on the surface of the connecting stems; (c) more or less uniform silica deposition at the surface of the paddle structures. Our Raman microscopy results suggest that; (d) silica is deposited in its amorphous form in thin layered structures; (e) there is a relative increase of silica at the sites of spherical containers; (f) pectin- and glucomannan-like glycosides may have some preference in the interior of the spherical containers; (g) the spherical containers are attached at the surface of paddle structures where cellulose-like planar bio-organic components dominate and where lignin contributions are diminished. The observed differences in distribution and character of inorganic and bio-organic structural elements in the elater of *Equisetum arvense* arise from the restrictions of prior morphogenesis and structural and elemental capacities gained during evolution to answer the practical challenges of initial proliferation and survival at the early stages of vegetation. To understand this, in Figure 9 are shown the SEM and optical microscopy images of spores on carbon tape 10 days after their deposition. These images show that the paddle structures undergo motion to interact with the carbon substrate with the consequence that some paddles and some spore central bodies demonstrate partial submerging into the carbon substrate. Further, some of the paddle structures became flattened and expanded and opening of spherical container to expose its contents to the environment is revealed (see Figure 9). The observed structural reorganization suggests that there exists an on-going morphogenesis and biochemistry which involves the elater paddles and spore central body to support effective germination. From these results, it can also be concluded that silica deposition at the elaters’ surface and their paddles as layered structures is beneficial for fast break down into a chemical substrate suitable for both, conditioning of local microbiology and to become ready for re-consumption as expected upon following the vegetation cycle. Opening of the internal spherical structures is likely to occur for the same purpose, while flattening and spreading of elaters may hinder photosynthesis of algae and other plants in the spatial region next to the spore central body.

Colloidal silica dissolution is a challenging task when applied to deposits in process cooling systems. Earlier studies suggested that hydroxyl ions play a catalytic role in the process (Jendoubi et al., 1997). However, later experimental studies indicated that an increase in the number of -COOH groups in various agents (capable to dissolve colloidal silica) does not have an obvious effect on dissolution efficiency. Instead, the presence of -PO_3_H_2_ and NH_2_ groups would appear to be important, and, particularly, under acidic environments (Mavredaki et al., 2005). Consequently, we may suggest that since degrading plant tissues typically generate an acidic environment (due to humid acid and other products), the side-group of arginine containing polysaccharide matrix (Perry, 1989) may facilitate the anticipated fast uptake of silica provided by the elater surface. This silica is particularly conditioned to: (i) not hinder the necessary flexibility and morphology of elaters; and, (ii) partition fast into the substrate in a colloidal form to be easily re-absorbed by young vegetation, facilitated by substrate chemistry pre-conditioned by decay and prior release of the content of the containers. Further, there are a range of opinions concerning whether silica may (Guerriero et al., 2018) or may not (Coskun et al., 2019) play a role in anti-fungal and anti-bacterial resistance mechanisms though it is not unreasonable to propose that fast re-absorption of silica would help improve the strength of structural elements within a living organism. If this is a skeletal component of a “few-cell” developing organism this may be along the biogenetic law stated by Haeckel of “ontogeneous recapitulation of phylogeny” (Haeckel, 1866). At the same time, we cannot exclude the opinion of the editor that fast silica re-absorption could be an evolutionary memory of mechanisms which are not obvious now but were possibly helpful in the past. It is possible that morphological and (bio)chemical studies of early *Equisetum* embryogenesis may help us understand if silica uptake was among the evolutionary advances of organisms (keeping in mind diatoms and sponges). This, however, is far beyond the scope of the current study.

## Conclusion

The use of sensitive Raman microscopy assisted with density functional theory is an attractive approach to explore the bio-mineral composition of *Equisetum* spore elaters spatially, both at the surface and within the biological structure. The spatial-spectral optical sampling correlates with structural properties detected using scanning electron microscopy. The approach suggests that silica is deposited in an amorphous, nearly colloidal form within structures that are up to a few layers thick that are readily dissolved and dispersed on germination. Silica and pectin-glucomannan-like glycosides may have some preference in the internal content of the spherical containers which are attached at the surface of paddle structures where a cellulose-like planar bio-organic component dominates and where lignin-like contributions are minimal. Spatial correlations of spectral signatures assist in addressing how structural properties and biochemical decoration of the elaters may support the physiology of the organelles and contribute to reproduction success.

## Author Contributions

CP and VV conceived the study. CP collected the plant material, performed the early studies on germination, and supervised the study. VV conducted the Raman microscopy and DFT calculations. GH conducted the SEM microscopy. AS-R conducted the material studies. All authors contributed in writing and reviewing the manuscript.

## Conflict of Interest Statement

The authors declare that the research was conducted in the absence of any commercial or financial relationships that could be construed as a potential conflict of interest.
