# Supplementary material for: Distributions of Silica and Biopolymer Structural Components in the Spore Elater of Equisetum arvense, an Ancient Silicifying Plant
Source: Front Plant Sci. 2019 Mar 5;10:210. doi: 10.3389/fpls.2019.00210 (PMC6412149; doi:10.3389/fpls.2019.00210)
Supplement: Supplementary file 1 [file Table_1.DOCX]

**SUPPORTING INFORMATION**

**Distributions of silica, carbohydrate and polysaccharide structural components in the spore elater of *Equisetum arvense*, an ancient silicifying plant**

Victor V. Volkov, Graham J. Hickman, Anna S. Rabada, Carole C. Perry^*^

*Interdisciplinary Biomedical Research Centre, School of Science and Technology, Nottingham Trent University, Clifton Lane, Nottingham NG11 8NS, United Kingdom*

Among the commons to United Kingdom species of *Equisetum* genus in *Equisetaceae*, such as *E. arvense* (Field Horsetail), *E. fluviatile* (Water Horsetail), *E. hyemale* (Rough Horsetail), *E. palustre* (Marsh Horsetail), *E. pratense* (Shade Horsetail), *E. ramosissimum* (Boston Horsetail), *E. sylvaticum* (Wood Horsetail), *E. telmateia* (Great Horsetail) and *E. variegatum* (Variegated Horsetail) we reserved our attention to structural and chemical properties of Field Horsetail (*E. arvense*), which is a common plant in England and in Nottinghamshire that inhabits meadows, gardens and wasteland. Field horsetail produces fertile spore bearing stems in March-April followed by green vegetative stems in late-spring. Its maximum vegetative growth occurs in July. We sampled the spore-heads of Field Horsetail along the river Leen in Lenton, Nottingham.


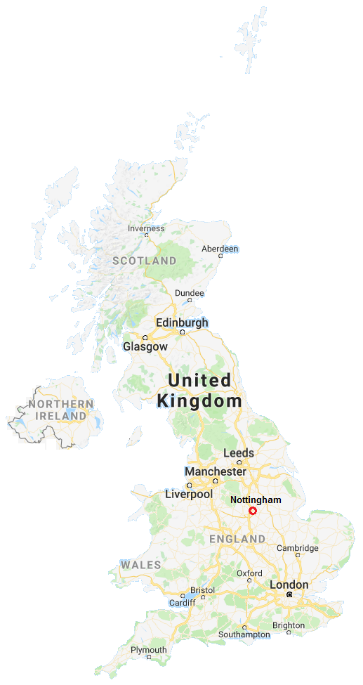

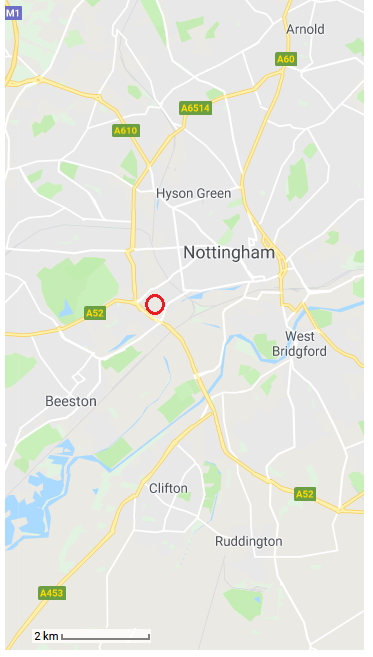

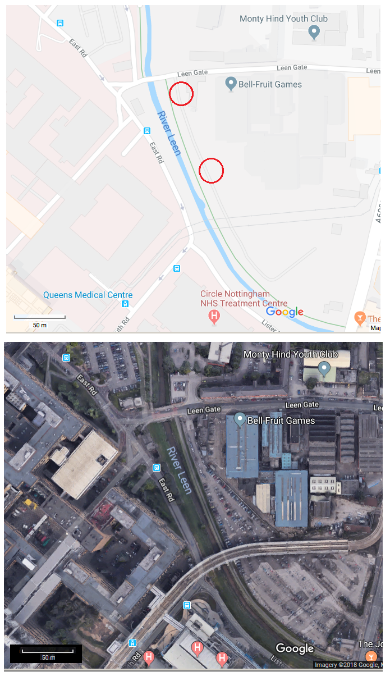

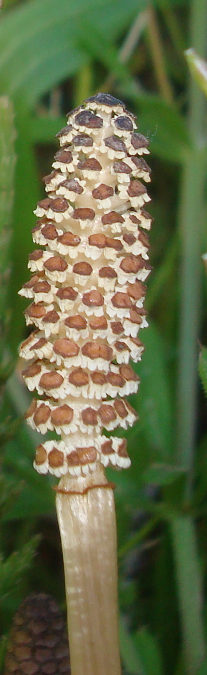


***Figure S1.*** *Sampling site of the spore-heads of Field Horsetail, E. arvense.*


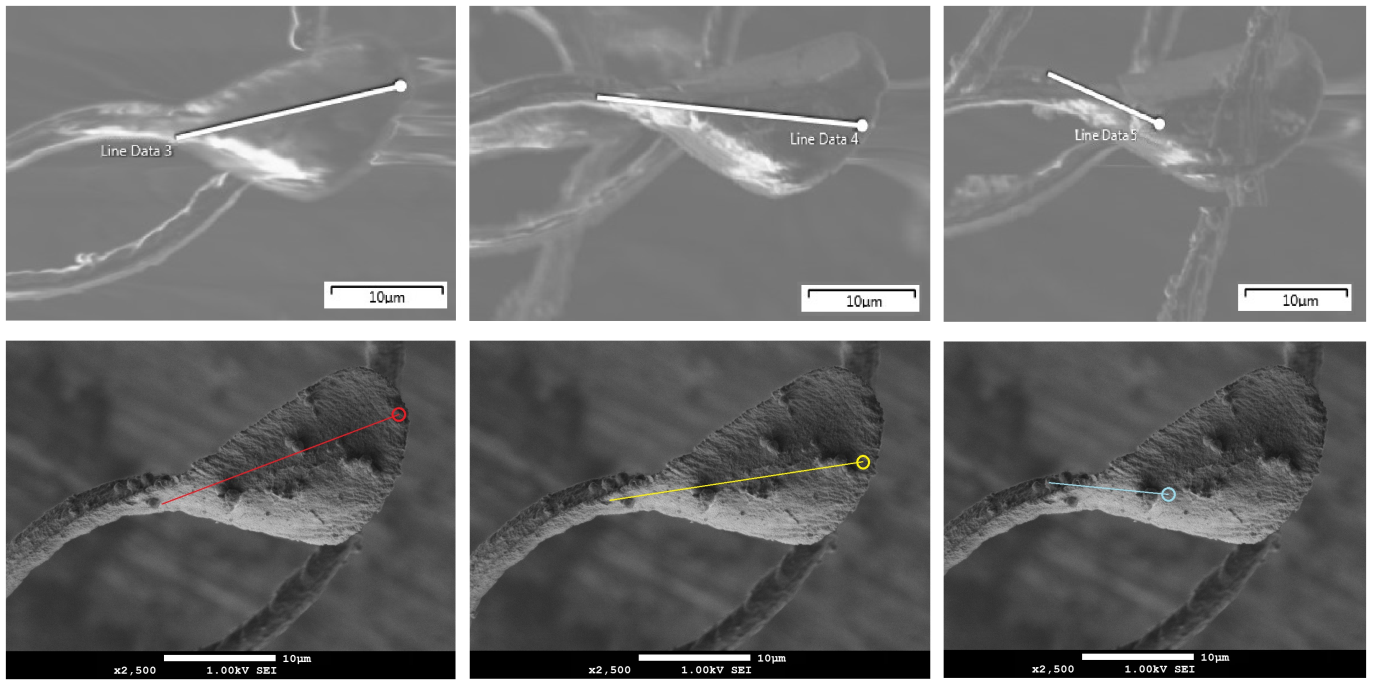


***Figure S2.*** *Top: original low-resolution SEM images of a selected elater with lines, along which the abundances of carbon, oxygen and silicon, using energy-dispersive X-ray spectroscopy, were sampled from right to left (circles mark the initial points). Bottom: high resolution SEM images of the same selected elater where we map the lines, along which the abundances of carbon, oxygen and silicon atoms were sampled, as shown in the top images.*

**
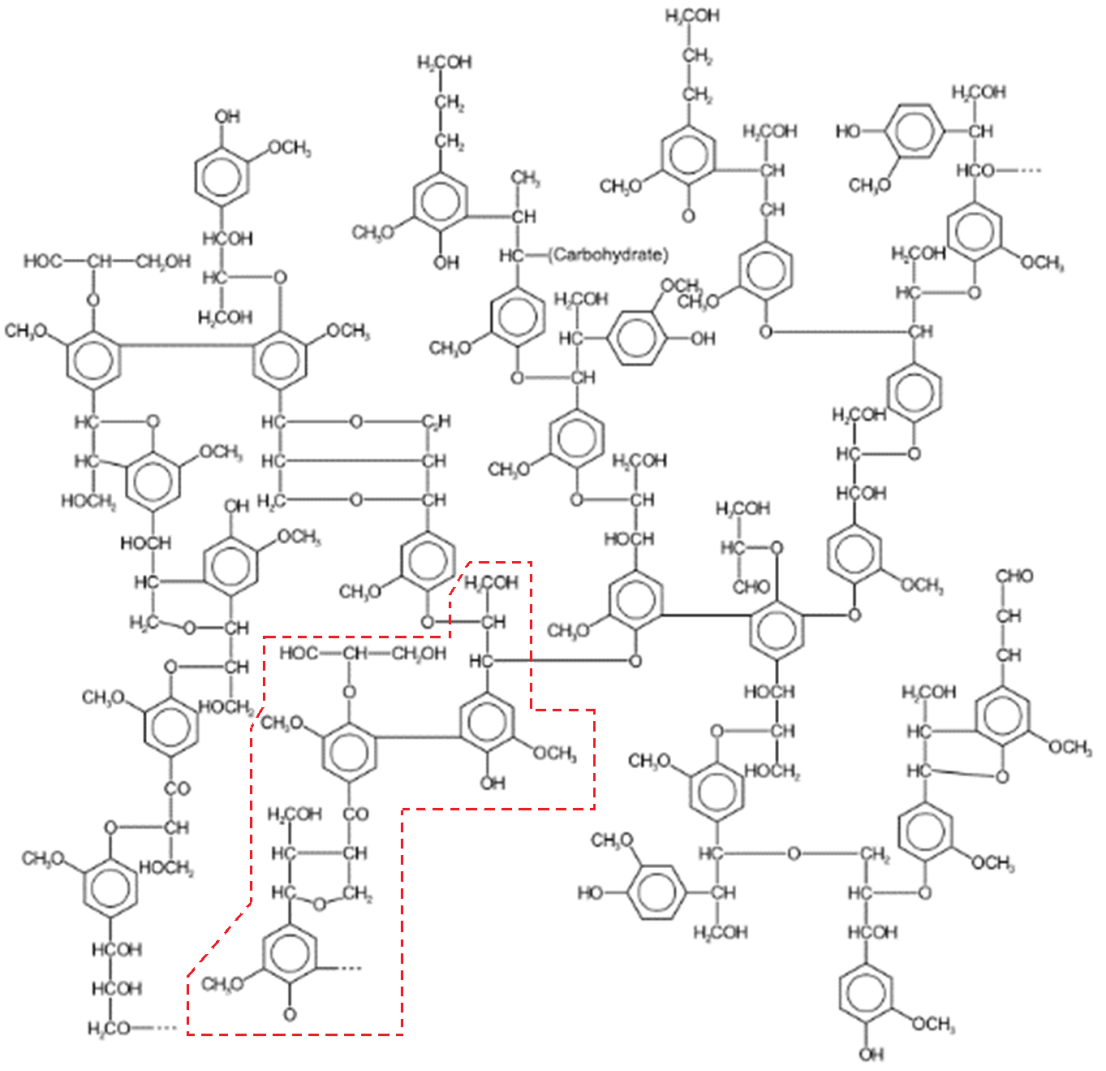
**

***Figure S3:*** *Segment of lignin (boxed by dashed red line) for DFT studies selected from the structure as discussed in Ref. [L. P. Christopher, B. Yao, Y. Ji. "Lignin biodegradation with laccase-mediator systems". Frontiers in Energy Research 2014, Vol. 2, 12:1-13.]*
